# Supplementary material for: Genomic Profiling of Sarcomas: A Promising Weapon in the Therapeutic Arsenal
Source: Int J Mol Sci. 2022 Nov 17;23(22):14227. doi: 10.3390/ijms232214227 (PMC9693140; doi:10.3390/ijms232214227)
Supplement: Supplementary file 1 [file ijms-23-14227-s001.zip › ijms-1974785-supplementary.pdf]

Supplementary Materials

Table S1. DNA gene list for the detection of base substitutions, insertion/deletions, and copy number alterations [1]

**DNA gene list: entire coding sequence for the detection of base substitutions, insertion/deletions, and copy number alterations.**				
ABL1	*CREBBP*	*GNAS*	*MEN1*	*RHOA*
ACTB	*CRKL*	*GPR124*	*MET*	*RICTOR*
AKT1	*CRLF2*	*GRIN2A*	*MIB1*	*RNF43*
AKT2	*CSF1R*	*GSK3B*	*MITF*	*ROS1*
AKT3	*CSF3R*	*GTSE1*	*MKI67*	*RPTOR*
ALK	*CTCF*	*HDAC1*	*MLH1*	*RUNX1*
AMER1 (FAM123B or WTX)	*CTNNA1*	*HDAC4*	*MPL*	*S1PR2*
APC	*CTNNB1*	*HDAC7*	*MRE11A*	*SDHA*
APH1A	*CUX1*	*HGF*	*MSH2*	*SDHB*
AR	*CXCR4*	*HIST1H1C*	*MSH3*	*SDHC*
ARAF	*DAXX*	*HIST1H1D*	*MSH6*	*SDHD*
ARFRP1	*DDR2*	*HIST1H1E*	*MTOR*	*SERP2*
ARHGAP26 (GRAF)	*DDX3X*	*HIST1H2AC*	*MUTYH*	*SETBP1*
ARID1A	*DNM2*	*HIST1H2AG*	*MYC*	*SETD2*
ARID2	*DNMT3A*	*HIST1H2AL*	*MYCL (MYCL1)*	*SF3B1*
ASMTL	*DOT1L*	*HIST1H2AM*	*MYCN*	*SGK1*
ASXL1	*DTX1*	*HIST1H2BC*	*MYD88*	*SMAD2*
ATM	*DUSP2*	*HIST1H2BJ*	*MYO18A*	*SMAD4*
ATR	*DUSP9*	*HIST1H2BK*	*NCOR2*	*SMARCA1*
ATRX	*EBF1*	*HIST1H2BO*	*NCSTN*	*SMARCA4*
AURKA	*ECT2L*	*HIST1H3B*	*NF1*	*SMARCB1*
AURKB	*EED*	*HNF1A*	*NF2*	*SMC1A*
AXIN1	*EGFR*	*HRAS*	*NFE2L2*	*SMC3*
AXL	*ELP2*	*HSP90AA1*	*NFKBIA*	*SMO*
B2M	*EP300*	*ICK*	*NKX2-1*	*SOCS1*
BAP1	*EPHA3*	*ID3*	*NOD1*	*SOCS2*
BARD1	*EPHA5*	*IDH1*	*NOTCH1*	*SOCS3*
BCL10	*EPHA7*	*IDH2*	*NOTCH2*	*SOX10*
BCL11B	*EPHB1*	*IGF1R*	*NPM1*	*SOX2*
BCL2	*ERBB2*	*IKBKE*	*NRAS*	*SPEN*
BCL2L2	*ERBB3*	*IKZF1*	*NSD2)*	*SPOP*
BCL6	*ERBB4*	*IKZF2*	*NT5C2*	*SRC*
BCL7A	*ERG*	*IKZF3*	*NTRK1*	*SRSF2*
BCOR	*ESR1*	*IL7R*	*NTRK2*	*STAG2*
BCORL1	*ETS1*	*INHBA*	*NTRK3*	*STAT3*
BIRC3	*ETV6*	*INPP4B*	*NUP93*	*STAT4*
BLM	*EXOSC6*	*INPP5D (SHIP)*	*NUP98*	*STAT5A*
BRAF	*EZH2*	*IRF1*	*P2RY8*	*STAT5B*
BRCA1	*FAF1*	*IRF4*	*PAG1*	*STAT6*

**DNA gene list: entire coding sequence for the detection of base substitutions, insertion/deletions, and copy number alterations.**				
BRCA2	FAM46C	IRF8	PAK3	STK11
BRD4	FANCA	IRS2	PALB2	SUFU
BRIP1	FANCC	JAK1	PASK	SUZ12
BRSK1	FANCD2	JAK2	PAX5	TAF1
BTG2	FANCE	JAK3	PBRM1	TBL1XR1
BTK	FANCF	JARID2	PC	TCF3 (E2A)
BTLA	FANCG	JUN	PCBP1	TCL1A (TCL1)
C11orf30 (EMSY)	FANCL	KAT6A (MYST3)	PCLO	TET2
CAD	FAS (TNFRSF6)	KDM2B	PDCD1	TGFBR2
CALR*	FBXO11	KDM4C	PDCD11	TLL2
CARD11	FBXO31	KDM5A	PDCD1LG2 (PD-L2)	TMEM30A
CBFB	FBXW7	KDM5C	PDGFRA	TMSB4XP8 (TMSL3)
CBL	FGF10	KDM6A	PDGFRB	TNFAIP3
CCND1	FGF14	KDR	PDK1	TNFRSF11A
CCND2	FGF19	KEAP1	PHF6	TNFRSF14
CCND3	FGF23	KIT	PIK3CA	TNFRSF17
CCNE1	FGF3	KLHL6	PIK3CG	TOP1
CCT6B	FGF4	KMT2A (MLL)	PIK3R1	TP53
CD22	FGF6	KMT2C (MLL3)	PIK3R2	TP63
CD274 (PD-L1)	FGFR1	KMT2D (MLL2)	PIM1	TRAF2
CD36	FGFR2	KRAS	PLCG2	TRAF3
CD58	FGFR3	LEF1	POT1	TRAF5
CD70	FGFR4	LRP1B	PPP2R1A	TSC1
CD79A	FHIT	LRRK2	PRDM1	TSC2
CD79B	FLCN	MAF	PRKAR1A	TSHR
CDC73	FLT1	MAFB	PRKDC	TUSC3
CDH1	FLT3	MAGED1	PRSS8	TYK2
CDK12	FLT4	MALT1	PTCH1	U2AF1
CDK4	FLYWCH1	MAP2K1	PTEN	U2AF2
CDK6	FOXL2	MAP2K2	PTPN11	VHL
CDK8	FOXO1	MAP2K4	PTPN2	WDR90
CDKN1B	FOXO3	MAP3K1	PTPN6 (SHP-1)	WHSC1 (MMSET or
CDKN2A	FOXP1	MAP3K14	PTPRO	WISP3
CDKN2B	FRS2	MAP3K6	RAD21	WT1
CDKN2C	GADD45B	MAP3K7	RAD50	XBP1
CEBPA	GATA1	MAPK1	RAD51	XPO1
CHD2	GATA2	MCL1	RAF1	YY1AP1
CHEK1	GATA3	MDM2	RARA	ZMYM3
CHEK2	GID4 (C17orf39)	MDM4	RASGEF1A	ZNF217
CIC	GNA11	MED12	RB1	ZNF24 (ZSCAN3)
CIITA	GNA12	MEF2B	RELN	ZNF703
CKS1B	GNA13	MEF2C	RET	ZRSR2
CPS1	GNAQ			

Table S2. DNA gene list for the detection of select rearrangements [1]

**DNA gene list: for the detection of select rearrangements**				
ALK	*EGFR*	*EWSR1*	*JAK2*	*RAF1*
BCL2	*EPOR*	*FGFR2*	*KMT2A (MLL)*	*RARA*
BCL6	*ETV1*	*IGH*	*MYC*	*RET*
BCR	*ETV4*	*IGK*	*NTRK1*	*ROS1*
BRAF	*ETV5*	*IGL*	*PDGFRA*	*TMPRSS2*
CCND1	*ETV6*	*JAK1*	*PDGFRB*	*TRG*
CRLF2				

Table S3. RNA gene list for the detection of select rearrangements [1]

**RNA gene list: for the detection of select rearrangements**				
ABI1	*CTNNB1*	*HOXC11*	*NF1*	*RPL22*
ABL1	*DDIT3*	*HOXC13*	*NF2*	*RPN1*
ABL2	*DDX10*	*HOXD11*	*NFKB2*	*RUNX1*
ACSL6	*DDX6*	*HOXD13*	*NIN*	*RUNX1T1 (ETO)*
AFF1	*DEK*	*HSP90AA1*	*NOTCH1*	*RUNX2*
AFF4	*DUSP22*	*HSP90AB1*	*NPM1*	*SEC31A*
ALK	*EGFR*	*IGH*	*NR4A3*	*SEPT5*
ARHGAP26 (GRAF)	*EIF4A2*	*IGK*	*NSD1*	*SEPT6*
ARHGEF12	*ELF4*	*IGL*	*NTRK1*	*SEPT9*
ARID1A	*ELL*	*IKZF1*	*NTRK2*	*SET*
ARNT	*ELN*	*IL21R*	*NTRK3*	*SH3GL1*
ASXL1	*EML4*	*IL3*	*NUMA1*	*SLC1A2*
ATF1	*EP300*	*IRF4*	*NUP214*	*SNX29 (RUNDC2A)*
ATG5	*EPOR*	*ITK*	*NUP98*	*SRSF3*
ATIC	*EPS15*	*JAK1*	*NUTM2A*	*SS18*
BCL10	*ERBB2*	*JAK2*	*OMD*	*SSX1*
BCL11A	*ERG*	*JAK3*	*P2RY8*	*SSX2*
BCL11B	*ETS1*	*JAZF1*	*PAFAH1B2*	*SSX4*
BCL2	*ETV1*	*KAT6A (MYST3)*	*PAX3*	*STAT6*
BCL3	*ETV4*	*KDSR*	*PAX5*	*STL*
BCL6	*ETV5*	*KIF5B*	*PAX7*	*SYK*
BCL7A	*ETV6*	*KMT2A (MLL)*	*PBX1*	*TAF15*
BCL9	*EWSR1*	*LASP1*	*PCM1*	*TAL1*
BCOR	*FCGR2B*	*LCP1*	*PCSK7*	*TAL2*
BCR	*FCRL4*	*LMO1*	*PDCD1LG2 (PD-L2)*	*TBL1XR1*
BIRC3	*FEV*	*LMO2*	*PDE4DIP*	*TCF3 (E2A)*
BRAF	*FGFR1*	*LPP*	*PDGFB*	*TCL1A (TCL1)*
BTG1	*FGFR1OP*	*LYL1*	*PDGFRA*	*TEC*
CAMTA1	*FGFR2*	*MAF*	*PDGFRB*	*TET1*
CARS	*FGFR3*	*MAFB*	*PER1*	*TFE3*
CBFA2T3	*FLI1*	*MALT1*	*PHF1*	*TFG*
CBFB	*FNBP1*	*MDS2*	*PICALM*	*TFPT*
CBL	*FOXO1*	*MECOM*	*PIM1*	*TFRC*
CCND1	*FOXO3*	*MKL1*	*PLAG1*	*TLX1*
CCND2	*FOXO4*	*MLF1*	*PML*	*TLX3*
CCND3	*FOXP1*	*MLLT1 (ENL)*	*POU2AF1*	*TMPRSS2*
CD274 (PD-L1)	*FSTL3*	*MLLT10 (AF10)*	*PPP1CB*	*TNFRSF11A*
CDK6	*FUS*	*MLLT3*	*PRDM1*	*TOP1*
CDX2	*GAS7*	*MLLT4 (AF6)*	*PRDM16*	*TP63*
CHIC2	*GLI1*	*MLLT6*	*PRRX1*	*TPM3*
CHN1	*GMPS*	*MN1*	*PSIP1*	*TPM4*
CIC	*GPHN*	*MNX1*	*PTCH1*	*TRIM24*
CIITA	*HERPUD1*	*MSI2*	*PTK7*	*TRIP11*
CLP1	*HEY1*	*MSN*	*RABEP1*	*TTL*
CLTC	*HIP1*	*MUC1*	*RAF1*	*TYK2*

RNA gene list: for the detection of select rearrangements

CLTCL1	*HIST1H4I*	*MYB*	*RALGDS*	*USP6*
CNTRL (CEP110)	*HLF*	*MYC*	*RAP1GDS1*	*WHSC1 (MMSET or NSD2)*
COL1A1	*HMGA1*	*MYH11*	*RARA*	*WHSC1L1*
CREB3L1	*HMGA2*	*MYH9*	*RBM15*	*YPEL5*
CREB3L2	*HOXA11*	*NACA*	*RET*	*ZBTB16*
CREBBP	*HOXA13*	*NBEAP1 (BCL8)*	*RHOH*	*ZMYM2*
CRLF2	*HOXA3*	*NCOA2*	*RNF213*	*ZNF384*
CSF1	*HOXA9*	*NDRG1*	*ROS1*	*ZNF521*

Table S4. Accuracy of FoundationOne® test. Adapted from [1]

**Accuracy of FoundationOne® Heme test**		
Sensitivity: Base Substitutions	At ≥5% Minor Allele Frequency	>99.0%
Sensitivity: Insertions/Deletions (1-40bp)	At ≥10% Minor Allele Frequency	98.0%
Sensitivity: Focal Copy Number Alterations (Homozygous Deletions or Amplifications)	At ≥8 copies	>95.0%
Sensitivity: Microsatellite status	At ≥20% tumor nuclei	97.0%
Sensitivity: Known Gene Fusions	>95.0%	
Specificity: Base Substitutions, Insertions/Deletions, and Focal Copy Number Alterations	Positive Predictive Value (PPV)	>99.0%
Specificity: Known Gene Fusions	Positive Predictive Value (PPV)	>95.0%
Specificity: Microsatellite status	Positive Predictive Value (PPV)	>95.0%
Accuracy: Tumor Mutation Burden	At ≥20% tumor nuclei	>90.0%
Reproducibility (average concordance between replicates)	97.0% inter-batch precision<br>97.0% intra-batch precision<br>95.0% microsatellite status precision 96.0% tumor mutation burden precision	

Reference

1. FoundationOne Medicine Sample Report - Acute Myeloid Leukemia. Availabe online: https://rochefoundationmedicine.com/F1Hemereport_AML (accessed on 02/11/2022).
